# Somatic symptoms in adolescence as a predictor of severe mental illness in adulthood: a long-term community-based follow-up study

**DOI:** 10.1186/s13034-018-0245-0

**Published:** 2018-08-14

**Authors:** Hannes Bohman, Sara B. Låftman, Neil Cleland, Mathias Lundberg, Aivar Päären, Ulf Jonsson

**Affiliations:** 1 Department of Neuroscience, Child and Adolescent Psychiatry, Uppsala University, Box 593, 75124 Uppsala, Sweden; 2 Department of Women’s and Children’s Health, Akademiska University Hospital, 75185 Uppsala, Sweden; 3 Department of Clinical Science and Education, Södersjukhuset/Karolinska Institutet, 11883 Stockholm, Sweden; 4 Department of Public Health Sciences, Stockholm University, 10691 Stockholm, Sweden; 5 Centre for Psychiatry Research, Department of Clinical Neuroscience, Karolinska Institutet, 17177 Stockholm, Sweden; 6 Department of Women’s & Children’s Health, Center for Neurodevelopmental Disorders at Karolinska Institutet (KIND), Karolinska Institutet, CAP Research Center, Gävlegatan 22B, Floor 8, 113 30 Stockholm, Sweden

## Abstract

**Background:**

Somatic symptoms are common and costly for society and correlate with suffering and low functioning. Nevertheless, little is known about the long-term implications of somatic symptoms. The objective of this study was to assess if somatic symptoms in adolescents with depression and in their matched controls predict severe mental illness in adulthood by investigating the use of hospital-based care consequent to different mental disorders.

**Methods:**

The entire school population of 16–17-year-olds in the city of Uppsala, Sweden, was screened for depression in 1991–1993 (n = 2300). Adolescents with positive screenings (n = 307) and matched non-depressed controls (n = 302) participated in a semi-structured diagnostic interview for mental disorders. In addition, 21 different self-rated somatic symptoms were assessed. The adolescents with depression and the matched non-depressed controls were engaged in follow-up through the National Patient Register 17–19 years after the baseline study (n = 375). The outcome measures covered hospital-based mental health care for different mental disorders according to ICD-10 criteria between the participants’ ages of 18 and 35 years.

**Results:**

Somatic symptoms were associated with an increased risk of later hospital-based mental health care in general in a dose–response relationship when adjusting for sex, adolescent depression, and adolescent anxiety (1 symptom: OR = 1.63, CI 0.55–4.85; 2–4 symptoms: OR = 2.77, 95% CI 1.04–7.39; ≥ 5 symptoms: OR = 5.75, 95% CI 1.98–16.72). With regards to specific diagnoses, somatic symptoms predicted hospital-based care for mood disorders when adjusting for sex, adolescent depression, and adolescent anxiety (p < 0.05). In adolescents with depression, somatic symptoms predicted later hospital-based mental health care in a dose–response relationship (p < 0.01). In adolescents without depression, reporting at least one somatic symptom predicted later hospital-based mental health care (p < 0.05).

**Conclusions:**

Somatic symptoms in adolescence predicted severe adult mental illness as measured by hospital-based care also when controlled for important confounders. The results suggest that adolescents with somatic symptoms need early treatment and extended follow-up to treat these specific symptoms, regardless of co-occurring depression and anxiety.

**Electronic supplementary material:**

The online version of this article (10.1186/s13034-018-0245-0) contains supplementary material, which is available to authorized users.

## Background

The experience of somatic symptoms, such as gastrointestinal pain, headache, back pain and tiredness, is common in the general population [1, 2]. Somatic symptoms are expensive in terms of direct costs for health care but also in a wider societal perspective due to decreased productivity [3, 4]. Research over the two past decades has documented that somatic symptoms are also common in community-based samples of children and adolescents, particularly among girls [5–7]. Children and adolescents suffering from somatic symptoms perform worse in school [8], are more often absent from school, and more often tend to have problematic social relations [9–11]. Somatic symptoms in children and adolescents are also associated with mental disorders such as anxiety and depression [9, 12–20] and with other severe concurrent psychiatric problems in a dose–response relationship—for example, conduct disorder, suicidal behavior, and experiences of multiple interpersonal conflicts [15, 21–23].

However, less is known about the long-term implications of somatic symptoms in childhood and adolescence and follow-up periods rarely stretch longer than until young adulthood. In particular, there is a lack of knowledge about the long-term outcomes of somatic symptoms when adjusted for concurrent mental disorders and other confounders [24]. Only a few studies have investigated the long-term interrelationship between somatic symptoms, depression and anxiety at both baseline and follow up [25, 26]. In addition, most of the previous long-term follow-up studies of somatic symptoms and later mental health outcomes have used self-reported measures of mental disorders at follow-up [24]. Thus, little is known about the potential severe implications of somatic symptoms in terms of, for example, the use of hospital-based mental health care.

In a previous study, we followed up on adolescents with depression and somatic symptoms until they reached an adult age. We showed that adolescents with somatic symptoms had increased risks of adult depression, anxiety and other mental disorders, independent of concurrent adolescent depression and other confounders [27]. Despite having important findings, the previous study suffered from some limitations. The study relied on self-reported interview diagnoses rather than on clinical diagnoses. Depression was recorded retrospectively, thus introducing the possibility of recall bias. Depression and somatic symptoms were assessed both at baseline and at follow up, but anxiety was not included in the baseline analyses in this study. In addition, in the previous study, we did not investigate the severity of the mental disorders, e.g., the use of advanced health care. In the present study, we use register data that included diagnoses of hospital-based mental health care during the 17- to 19-year follow-up period. These data enabled us to investigate severe mental illness in terms of the use of advanced health care for mental disorders without the possibility of recall bias. The data also allowed us to assess the predictive power of somatic symptoms in adolescence, while adjusting for depression and anxiety in adolescence as well as sex and other potential confounders.

The aim of the current study was to test the hypothesis that adolescent somatic symptoms predict severe mental illness in adulthood. We address three research questions:

1. Are somatic symptoms in adolescents a predictor for later severe mental illness, measured by the use of adult hospital-based care for mental disorders, while also adjusting for adolescent depression and anxiety and other important confounders?
2. Are the number of concurrent somatic symptoms in depressed adolescents a predictor for later severe mental illness, measured by the use of adult hospital-based care for mental disorders?
3. Are somatic symptoms in non-depressed adolescents a predictor for later severe mental illness, measured by the use of adult hospital-based care for mental disorders?

## Methods

### Study population and procedure

**Fig. 1:**
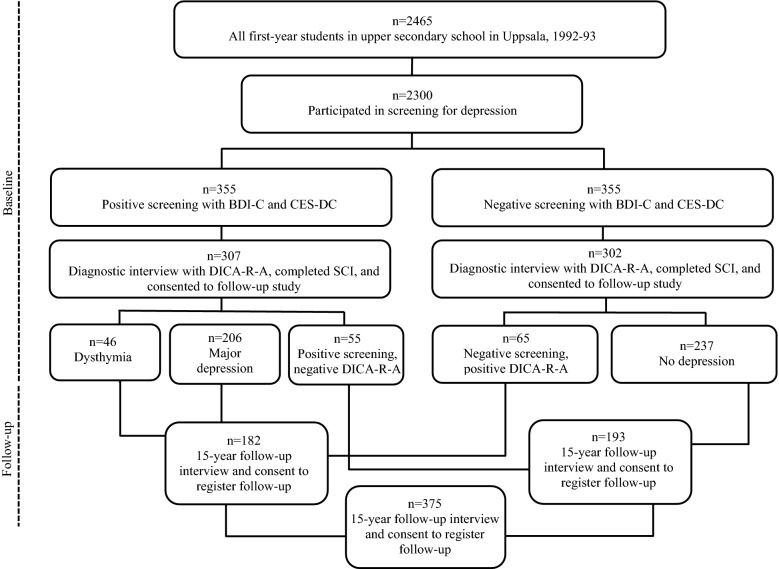
Chart outlining the data-collection procedure at baseline (in adolescence) and at follow-up (in adulthood)

In 1991–1993, all first-year students in upper secondary school (16–17 years old) in the Swedish university town of Uppsala, with approximately 180,000 inhabitants, were asked to participate in a screening for depression [28]. School dropouts were also invited. Out of a total of 2465 individuals, 93% (n = 2300) participated in the screening, which included two self-evaluations of depression: the Beck Depression Inventory-Child and the Centre for Epidemiological Studies-Depression Scale for Children [29]. Students with high scores and those who reported a suicide attempt were interviewed with the Diagnostic Interview for Children and Adolescents with a revised form according to the DSM-III-R (DICA-R-A) [30]. In all, 355 students in the screening were classified as suffering from depression and were accordingly selected for a diagnostic interview. For each depressed student, a same-sex class-mate and with low scores in the screening was recruited into a comparison group. In total, 609 individuals (n = 307 in the depressed group and n = 302 in the control group) participated in the diagnostic interview and consented to be contacted for a future follow-up study. At the time of the interview, they also completed a range of self-rating measures, including the Somatic Symptom Checklist Instrument (SCI) on somatic symptoms. Some of the participants in the comparison group (n = 65) were retrospectively diagnosed with major depression or dysthymia occurring before the baseline study and consequently were included in the depression group. Some of the participants with positive screenings did not meet the criteria for a depressive disorder upon being interviewed for current and lifetime major depression or dysthymia and were in the present analyses relocated to the control group (n = 55). Approximately 15 years after the baseline study, the participants who had consented to a follow-up study were contacted and invited to a follow-up interview. They were also asked if they wanted to participate in studies that included health registers. Data were subsequently collected from health registers 17–19 years after the baseline study. Among the 609 individuals who had participated in the diagnostic interview and who also had completed the SCI at baseline, approximately 70% participated in the follow-up interview. Of these, 375 individuals gave their written consent to be followed through the health registers (n = 182 in the depression group and n = 193 in the control group). The procedure is outlined in Fig. 1. Further information about the follow-up study is provided elsewhere [27, 31].

### Adolescent depression

Adolescent depression was defined as major depressive disorder (MDD) or dysthymia according to DICA-R-A [30] (see Fig. 1).

### Adolescent anxiety

Adolescent anxiety was defined as any anxiety disorder according to DICA-R-A [30].

### Adolescent somatic symptoms

The SCI is a Swedish version of the Psychosomatic Symptom Checklist [32]. The SCI assesses 22 items reflecting various somatic symptoms: tiredness, headache, feeling chilly, insomnia, eye tiredness, abdominal pain, dizziness, nausea, perspiration, appetite problem, breathing problem, polyuria, limb pain, itching, dry mouth, palpitation, constipation, fainting, regurgitation, chewing pain, and swallowing problems. Allergy was part of the checklist but was excluded from the analyses because it was considered a somatic disease rather than a symptom. The symptoms were graded in frequency (0 = never, 1 = monthly, 2 = weekly, 3 = several times a week, and 4 = daily), and intensity (0 = no problem, 1 = minor, 2 = moderate, 3 = troublesome, and 4 = extremely troublesome), for the last month. The questionnaire has been used in previous publications [12, 23, 27]. A somatic symptom was recorded when the frequency and intensity were multiplied to yield a score ≥ 6 (e.g., 2 × 3: weekly × troublesome symptoms). Such a scoring approach excluded minor problems and the possibility that monthly premenstrual symptoms would be recorded as positive. The same cut-off has been used in earlier publications [23, 27].

In the analyses of the control group, somatic symptoms were categorized as 0 vs. ≥ 1 symptoms (a more fine-grained categorization was not possible due to small numbers in the cells). In the analyses of individuals with adolescent depression, four categories of somatic symptoms were created: 0, 1, 2–4, and ≥ 5 symptoms—a categorization that was grounded in our previous analyses of the same data material, where ≥ 5 somatic symptoms were found to characterize a threshold value in the prediction of mental health outcomes in adulthood [27].

### Confounders

A set of potential confounders, which may potentially have affected both somatic symptoms at baseline and mental disorders in adulthood, were used to adjust the analyses. Information on conflicts between parents, conflicts with parents, economic hardship, parental unemployment, and somatic illness collected at baseline through the Children’s Life Inventory [33] was included. In addition, we included information on physical/sexual abuse in childhood collected retrospectively in the follow-up study [31]. Conflicts between parents and conflicts with parents were shown to be significantly related to major depression at baseline [34] as well as to somatic symptoms at baseline [23], and analyses of the follow-up data demonstrated that conflicts with parents and physical/sexual abuse in childhood were associated with mental disorders in adulthood [31]. Socioeconomic status in the family of origin had been shown to be associated with mental disorders in adulthood [35]; therefore, measures of economic hardship and parental unemployment collected at baseline were included. In order to account for the fact that some somatic symptoms might have had a medical explanation (i.e. due to somatic illness), a measure of somatic illness reported in adolescence was included. The variable was created from two items from the Children’s Life Inventory [33]: “I have been severely ill or injured”, and “I have been hospitalized more than one week”, with the possible response categories “During the past year” and “Earlier in life”. The measure of somatic illness was defined by a positive record on at least one of these two items, i.e., self-reported somatic illness or injury some time in life until baseline and/or the adolescent’s report on having ever been hospitalized more than 1 week some time in life until baseline. The data did however not include any information about specific somatic diagnoses.

### Outcomes

The Swedish National Health and Welfare Board maintains the official registers concerning health and sickness in Sweden. The national patient register was used in the present study from 1992 until 2009. The national patient register includes data on inpatient care and outpatient hospital-based care. With regard to inpatient care, the register data cover almost all inpatient visits since 1987. With regard to hospital-based outpatient care, outpatient visits have been registered since 2001, but only a part of the data is covered during the follow-up period. Hospital-based mental health care diagnoses were classified according to ICD-10 criteria—specifically, the codes F10–F69 were used to define hospital-based mental health care. For more detailed analyses, the diagnoses were also divided into different general categories: F10–F19, mental and behavioral disorders due to psychoactive substance use; F20–F29, schizophrenia, schizotypal and delusional disorders; F30–F39, mood disorders; F40–F48, neurotic, stress-related and somatoform disorders (including all anxiety disorders); F50–F59, behavioral syndromes associated with physiological disturbances and physical factors; and F60–F69, disorders of adult personality and behavior.

### Data analysis

Binary logistic regression analyses were performed to assess the association of somatic symptoms in adolescence with later hospital-based mental health care. Adjustments were made for adolescent depression and anxiety, sex and other potential confounders. Odds ratios with 95% confidence intervals were reported. In the descriptive analyses of somatic symptoms and specific mental health care diagnoses, when several categories of somatic symptoms were compared, linear-by-linear associations were used to calculate linear relationships. To compare the groups of individuals with 0 and ≥ 1 somatic symptoms at baseline, respectively, the Fisher’s exact test was used. Stata version 15 (StataCorp, College Station, TX) was used.

## Results

Descriptive statistics for the pooled sample and, separately, for individuals without and with adolescent depression are presented in Table 1. Adolescents with depression had more concurrent somatic symptoms on average compared to the controls (3.10 vs. 1.27, p < 0.001). (Details on the prevalence of specific somatic symptoms are provided in Additional file 1: Appendix S1). All of the included potential confounders were substantially more common among individuals with adolescent depression than among controls without adolescent depression. In adulthood, any hospital-based mental health care diagnosis was significantly more common in the depressed group than in the control group (OR = 2.80, p < 0.01). This pattern was reflected in all specific diagnoses, although the difference between groups was statistically significant only for mood disorders. As seen in Table 1, however, when distinguishing any hospital-based mental health care at the level of the specific diagnosis, the absolute numbers of cases were small.

**Table 1:** Descriptive statistics for the pooled sample and separately for adolescents without depression (control group) and adolescents with depression at baseline, and differences between these groups (reference category = control group)

	All (n = 375)	Adolescents without depression (n = 182)	Adolescents with depression (n = 193)	OR	95% CI
	% (n)	% (n)	% (n)		
Sex					
Males (ref.)	19.7 (74)	22.0 (40)	17.6 (34)	1.00	–
Females	80.3 (301)	78.0 (142)	82.4 (159)	1.32	0.79–2.19

	Mean (s.d.)	Mean (s.d.)	Mean (s.d.)	*t* test	
Adolescence					
Number of concurrent somatic symptoms	2.21 (2.36)	1.27 (1.76)	3.10 (2.50)	p < 0.001	

	Mean (s.d.)	Mean (s.d.)	Mean (s.d.)	χ2	p
0	27.5 (103)	41.7 (76)	14.0 (27)		
1	23.2 (87)	26.4 (48)	20.2 (39)		
2–4	34.4 (129)	27.5 (50)	40.9 (79)		
≥5	14.9 (56)	4.4 (8)	24.9 (48)	59.06	< 0.001

	% (n)	% (n)	% (n)	OR	95% CI
Adolescent anxiety	28.0 (105)	7.1 (13)	47.7 (92)	11.84***	6.30–22.25
Conflicts between parents	20.8 (78)	11.5 (21)	29.5 (57)	3.21***	1.85–5.57
Conflicts with parents	19.5 (73)	7.7 (14)	30.6 (59)	5.28***	2.83–9.87
Physical abuse	12.3 (46)	6.0 (11)	18.1 (35)	3.44**	1.69–7.01
Economic hardship	6.9 (26)	1.7 (3)	11.9 (23)	8.07**	2.38–27.38
Parental unemployment	11.5 (43)	6.6 (12)	16.1 (31)	2.71**	1.35–5.46
Somatic illness	20.3 (76)	13.7 (25)	26.4 (51)	2.26**	1.33–3.83

	% (n)	% (n)	% (n)	OR	95% CI
Adulthood					
Any hospital-based mental health care diagnosis	15.2 (57)	8.8 (16)	21.2 (41)	2.80**	1.51–5.19
F10–F19 Mental and behavioral disorders due to psychoactive substance use	2.1 (8)	1.7 (3)	2.6 (5)	1.59	0.37–6.74
F20–F29 Schizophrenia, schizotypal and delusional disorders	0.5 (2)	0.0 (0)	1.0 (2)	–	–
F30–F39 Mood disorders	7.2 (27)	4.4 (8)	9.8 (19)	2.38*	1.01–5.57
F40–F48 Neurotic, stress-related and somatoform disorders	9.1 (34)	6.6 (12)	11.4 (22)	1.82	0.87–3.80
F50–F59 Behavioral syndromes associated with physiological disturbances and physical factors	1.9 (7)	1.7 (3)	2.1 (4)	1.26	0.28–5.72
F60–F69 Disorders of adult personality and behavior	1.6 (6)	1.1 (2)	2.1 (4)	1.90	0.34–10.53

*** p < 0.001, ** p < 0.01, * p < 0.05

In a series of binary logistic regression analyses in the pooled sample of individuals with and without adolescent depression, the association between somatic symptoms in adolescence and adult hospital-based mental health care was analyzed (Table 2). The crude model included only the categories of somatic symptoms, showing that the number of somatic symptoms was associated with any hospital-based mental health care in a step-wise manner (for 2–4 symptoms OR = 3.51, 95% CI 1.37–8.98, and for ≥ 5 somatic symptoms OR = 8.30, 95% CI 3.08–22.41). Model 1 added adolescent depression, adolescent anxiety, and sex. The estimates for the categories of somatic symptoms were attenuated, but those corresponding to 2–4 and ≥ 5 somatic symptoms remained robust and statistically significant (OR = 2.77, 95% CI 1.04–7.39, and OR = 5.75, 95% CI 1.98–16.72, respectively). Model 2 added a number of potential confounders measured in adolescence, i.e., conflicts between parents, conflicts with parents, physical abuse, economic hardship, and parental unemployment. The association between ≥ 5 somatic symptoms and any hospital-based mental health care diagnosis in adulthood remained robust and statistically significant (OR = 5.03, 95% CI 1.66–15.28). To test whether the association between somatic symptoms in adolescence and hospital-based mental health care diagnosis in adulthood differed between adolescents with and without depression, an interaction term between somatic symptoms and adolescent depression was included. This was however not shown to be statistically significant (p = 0.587). Furthermore, to assess whether certain somatic symptoms were especially powerful predictors of later hospital-based mental health care, we also performed analyses of the associations between each specific somatic symptom and hospital-based mental health care in adulthood. Those that turned out to be statistically significant were tiredness, insomnia, headache, limb pain, abdominal pain, nausea and perspiration without exercise (see Additional file 1: Appendix S2).

**Table 2:** Odds ratios and 95% confidence intervals from binary logistic regression analyses of any hospital-based mental health care diagnosis in the pooled sample, n = 375

	n	Crude^a^		Model 1^b^		Model 2^c^	
		OR	95% CI	OR	95% CI	OR	95% CI
Number of somatic symptoms							
0 (ref.)	103	1.00	–	1.00	–	1.00	–
1	87	1.87	0.64–5.47	1.63	0.55–4.85	1.54	0.51–4.66
2–4	129	3.51**	1.37–8.98	2.77*	1.04–7.39	2.67	0.98–7.25
≥5	56	8.30***	3.08–22.41	5.75**	1.98–16.72	5.03**	1.66–15.28

^a^Crude includes categories pertaining to the number of somatic symptoms

^b^Model 1 adds adolescent depression, adolescent anxiety, and sex

^c^Model 2 adds conflicts between parents, conflicts with parents, physical abuse, economic hardship, parental unemployment, and somatic illness

*** p < 0.001, ** p < 0.01, * p < 0.05

Next, we present analyses of the associations between somatic symptoms and specific psychiatric diagnoses. As reported in Table 1, multiple somatic symptoms were more common among adolescents with depression than among those without depression. Therefore, for adolescents with depression we performed analyses of the number of somatic symptoms and psychiatric diagnoses (presented in Table 3), whereas for adolescents without depression we assessed the association between the presence of any (≥ 1) somatic symptom and psychiatric diagnoses (presented in Table 4).

**Table 3:** Adult hospital-based mental health care diagnoses at follow-up among individuals with adolescent depression, respectively, and numbers of somatic symptoms

Number of somatic symptoms	Individuals with adolescent depression (n = 193)				
	0 (n = 27)	1 (n = 39)	2–4 (n = 79)	≥5 (n = 48)	Linear by linear
	% (n)	% (n)	% (n)	% (n)	
Any hospital-based mental health care diagnosis	14.8 (4)	10.3 (4)	19.0 (15)	37.5 (18)	*p *< *0.01*
F10–F19 Mental and behavioral disorders due to psychoactive substance use	3.7 (1)	0.0 (0)	1.3 (1)	6.3 (3)	n.s.
F20–F29 Schizophrenia, schizotypal and delusional disorders	0.0 (0)	0.0 (0)	1.3 (1)	2.1 (1)	n.s.
F30–F39 Mood disorders	0.0 (0)	2.6 (1)	11.4 (9)	18.8 (9)	*p *< *0.01*
F40–F48 Neurotic, stress-related and somatoform disorders	11.1 (3)	7.7 (3)	7.6 (6)	20.8 (10)	n.s.
F50–F59 Behavioral syndromes associated with physiological disturbances and physical factors	0.0 (0)	2.6 (1)	3.8 (3)	0.0 (0)	n.s.
F60–F69 Disorders of adult personality and behavior	0.0 (0)	0.0 (0)	2.5 (2)	4.2 (2)	n.s.

**Table 4:** Adult hospital-based mental health care diagnoses at follow-up among individuals without adolescent depression, and numbers of somatic symptoms

Number of somatic symptoms	Individuals without adolescent depression (n = 182)		
	0 (n = 76)	≥1 (n = 106)	Fisher’s exact test
	% (n)	% (n)	
Any hospital-based mental health care diagnosis	2.6 (2)	13.2 (14)	*p *< *0.05*
F10–F19 Mental and behavioral disorders due to psychoactive substance use	0.0 (0)	2.8 (3)	n.s.
F20–F29 Schizophrenia, schizotypal and delusional disorders	0.0 (0)	0.0 (0)	–
F30–F39 Mood disorders	1.3 (1)	6.6 (7)	n.s.
F40–F48 Neurotic, stress-related and somatoform disorders	1.3 (1)	10.4 (11)	*p *< *0.05*
F50–F59 Behavioral syndromes associated with physiological disturbances and physical factors	0.0 (0)	2.8 (3)	n.s.
F60–F69 Disorders of adult personality and behavior	0.0 (0)	1.9 (2)	n.s.

Among individuals with adolescent depression, the likelihood of having received any hospital-based mental health care was associated with somatic symptoms in a linear manner (p < 0.01) (Table 3). Among the specific diagnoses, a statistically significant linear relationship with the number of somatic symptoms was only found for mood disorders (p < 0.01). Yet, for nearly all specific diagnoses (except for behavioral syndromes), hospital-based mental health care was most prevalent in the category with five or more somatic symptoms.

The presence of adult hospital-based mental health care among individuals without adolescent depression (i.e., the controls), differentiated by the presence of somatic symptoms in adolescence, is presented in Table 3. Compared with the controls without somatic symptoms, those with ≥ 1 somatic symptoms were more likely to have received hospital-based mental health care in adulthood (2.6% vs. 13.2%, respectively; p < 0.05). Among the specific diagnoses, hospital-based care for neurotic, stress-related and somatoform disorders (1.3% vs. 10.4%; p < 0.05) differed significantly between the controls without and with one or more somatic symptoms in adolescence.

Next, we wanted to compare the strength of association of somatic symptoms, depression, and anxiety, respectively, with mood disorders and for neurotic, stress-related and somatoform disorders at follow-up.

**Fig. 2:**
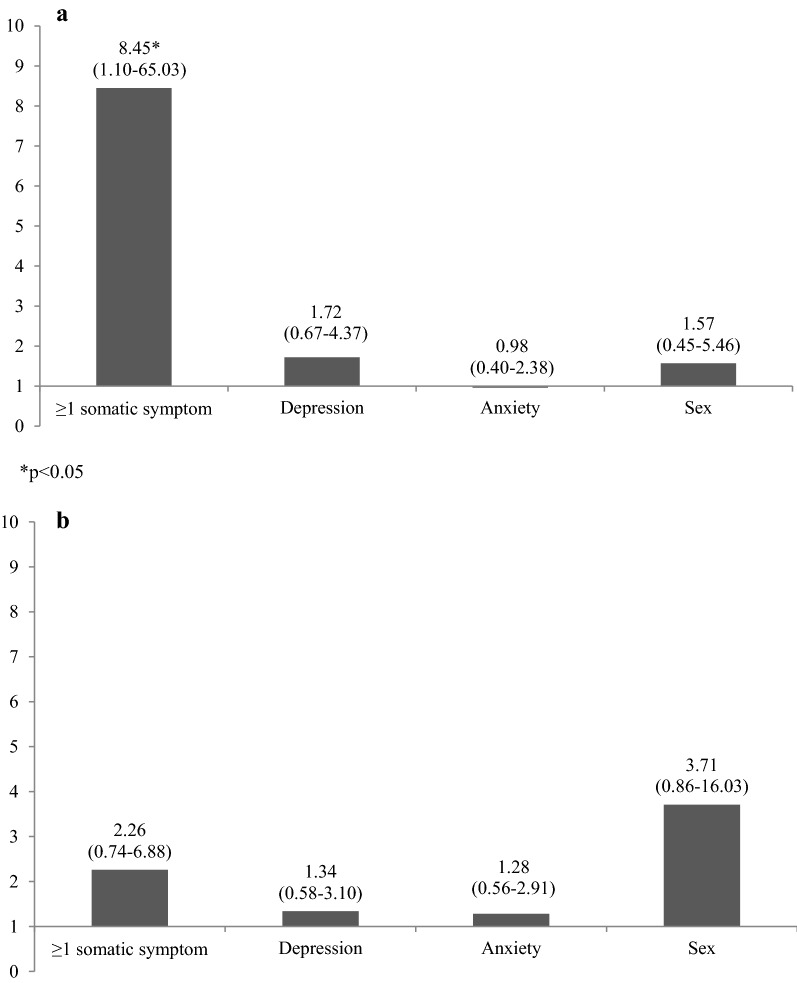
**a** Odds ratios with 95% confidence intervals from a binary logistic regression of hospital-based mental health care for mood disorders in the pooled sample, mutually adjusting for ≥ 1 somatic symptom, adolescent depression, adolescent anxiety, and sex, n = 375. **b** Odds ratios with 95% confidence intervals from a binary logistic regression of hospital-based mental health care for neurotic, stress-related and somatoform disorders in the pooled sample, mutually adjusting for ≥ 1 somatic symptom, adolescent depression, adolescent anxiety, and sex, n = 375

Figure 2a presents odds ratios from a binary logistic regression analysis of mood disorders. In the analysis, mutual adjustments were made for somatic symptoms, sex, and depression and anxiety in adolescence. The presence of ≥ 1 adolescent somatic symptom was a particularly strong predictor of adult hospital-based mental health care due to mood disorders (OR = 8.45, 95% CI 1.10–65.03), when mutually adjusting for sex, depression and anxiety in adolescence. When adjusting for the full set of confounders (i.e. adding also conflicts between and with parents, physical abuse, economic hardship, parental unemployment and somatic illness), the estimate was somewhat attenuated and turned non-significant (OR = 7.06, 95% CI 0.90–55.33, p = 0.063) (analysis not presented). Since the number of individuals with mood disorders was small, especially among those who did not report any somatic symptoms, this finding should however be interpreted with caution.

Figure 2b presents odds ratios from a binary logistic regression analysis of neurotic, stress-related and somatoform disorders. Somatic symptoms were not a significant predictor of neurotic, stress-related and somatoform disorders (OR = 2.26, 95% CI 0.74–6.88). Results from analyses including the full set of confounders (not presented) showed a similar pattern (OR = 2.12, 95% CI 0.68–6.61).

## Discussion

This study demonstrated that somatic symptoms in adolescence were associated with long-term severe mental health problems insofar as somatic symptoms did predict adult hospital-based mental health care in adulthood. For individuals with adolescent depression, there was a linear association between the number of somatic symptoms and later use of hospital-based mental health care. For individuals without adolescent depression, any somatic symptom was predictive of later use of hospital-based mental health care.

The findings that somatic symptoms independently predicted later mental health problems reflect those of a previous study using the same baseline data but with follow-up information on depression in adulthood from diagnostic interviews instead of register data on hospital-based mental health care [27]. Thus, the patterns were similar irrespective of whether the mental disorders were captured through interview or through diagnoses in clinical settings, implying that the findings are robust. The results are also in line with two recent American studies. Shanahan et al. [25] investigated abdominal pain, muscular pain, and headache with several assessments between 9 and 16 years and anxiety and depression in early adulthood, measured by diagnostic interviews. They found that frequent and recurrent somatic symptoms in childhood predicted anxiety and depression in adulthood after controlling for adolescent anxiety and depression as well as other potential confounders. Shelby et al. [26] found a prediction of functional abdominal pain in childhood and anxiety and depression until young adulthood. By analyzing hospital-based mental health care diagnoses as outcome measures, the current study corroborates the findings of these earlier studies but also extends them by demonstrating that somatic symptoms—in addition to implying risk of developing depression later in life—also predict a long-term risk of severe mental illness. Furthermore, the results indicate that somatic symptoms might not be less severe than established mental disorders, such as depression and anxiety, in terms of future mental health outcomes and could be an important target for treatment and prevention.

Earlier cross-sectional studies have shown that multiple somatic symptoms are associated with an increased risk of depression as well as depression severity among adolescents in a dose–response relationship [23]. The current study showed that a dose–response relationship also characterizes the long-term risk of hospital-based mental health care, with a particular high risk connected to a high number of somatic symptoms (≥ 5).

Not merely several somatic symptoms but even the presence of few were associated with the outcome in this study. Notably, among the non-depressed adolescents, having one or more somatic symptoms compared to none was associated with a significantly increased risk of later hospital-based mental health care. It should however be noted that while milder symptoms are relatively common even in non-depressed adolescents, in the present study we focused on symptoms with higher severity (as captured through their frequency and intensity).

Furthermore, there might be a stronger link between somatic symptoms and mood disorders than between somatic symptoms and other mental health diagnoses. Having one or more somatic symptoms compared to no somatic symptoms in adolescence predicted hospital-based care of mood disorders better than adolescent depression and anxiety when mutually adjustments were made. The prediction of hospital-based care for anxiety and somatoform disorders did however not reach statistical significance when adjusting for adolescent depression, anxiety and sex.

The finding that different somatic symptoms were an independent predictor of future hospital-based care of mood disorders has, to our knowledge, not been previously reported, although a Finnish population-based study found that abdominal pain in childhood predicted severe suicidal behavior (suicide and hospital care for suicidal attempts) among men [36].

The mechanisms that link somatic symptoms with future use of hospital-based mental health care for depressive and other disorders might involve different processes. Adolescents with somatic symptoms might have an increased help-seeking behavior which could explain their increased use of hospital-based mental health care in adulthood. Yet, the results from a previous study based on the same data material with adult depression diagnoses based on interviews shows the same pattern, namely, that somatic symptoms predict mental disorders independent from depression and other confounders [27]. This finding speaks against the possibility that help-seeking behavior is an important mediator in the association between somatic symptoms and later hospital-based mental health care. Somatic symptoms could also precipitate unhealthy living conditions that ultimately increase the risk of severe mental illness. For instance, individuals suffering from somatic symptoms might more often fail in higher education [37], and higher education is protective against adverse health outcomes [38]. The link between somatic symptoms and later hospital-based mental health care might also involve biological processes. Both somatic symptoms and emotional distress disorders include dysregulation of the HPA axis and serotonergic pathways [39]. Somatic symptoms could also involve the cytokine system, which underlies inflammatory-based pathways to emotional distress disorders [40]. However, whether there is a dose–response relationship between the number of somatic symptoms and biological markers remains to be shown. Furthermore, somatic symptoms (in particular abdominal pain) could hypothetically be indicative of maladaptive function of the gut involving microbiota, which, in turn, may be involved in regulating physiological systems important in emotional distress disorders [41].

Somatic symptoms have often been regarded as mental disorders by exclusion, as was the case in DSM-IV (but not in DSM-5) for somatoform disorders/somatic symptom disorders [42]. Due to an exclusion of other medical conditions, somatic symptoms might have been regarded as being caused by underlying psychological problems and therefore might not have been the focus of treatment. One implication of such earlier theories might have been a low priority of developing and disseminating effective treatment for somatic symptoms, especially when other problems such as depression and anxiety co-occur. Yet, the long-term implications of adolescent somatic symptoms presented in this study indicate that the treatment of somatic symptoms should have a higher priority in mental health services, particularly because emerging data indicate that treatment can be effective [43, 44].

### Strengths and limitations

The data and materials had several strengths. The baseline data were population-based, including 2300 adolescents of the same age, with a high participation rate (93%) in the depression screening. Another advantage was the long follow-up period from adolescence to adulthood. The prospective study design and the use of register data enabled us to follow individuals over time and to avoid the problem of recall bias. The data also provided the opportunity to investigate mental disorders and somatic symptoms at both baseline and follow-up (although neurotic, stress-related and somatoform disorders at follow-up were grouped together). A limitation was that only about two-thirds of participants in the original investigation were included in the present register-based follow-up. Yet, the participation rate can be seen as reasonably high in relation to the follow-up period. Furthermore, the attrition rates at follow-up were similar between the depressed and control groups. We assessed bivariate associations between somatic symptoms and later hospital-based mental health care in the two groups separately. To investigate the prediction of somatic symptoms whilst also including a set of potential confounders, we also performed analyses of the pooled sample. This design has limitations since the groups of depressed adolescents and their non-depressed matched peers were different in several respects, as shown in Table 1. Since only a fraction of non-depressed adolescents were included in the data, the pooled sample is not representative of the original population of 16–17-year-olds in the city of Uppsala. Yet, when assessing the relationship between adolescent somatic symptoms and later hospital-based mental health care, it is of high relevance to control not only for adolescent depression but also for anxiety and other confounders and in this study, this required a pooled sample.

In the present study, we chose to focus on severe mental illness and not on total consumption of mental health care. We did not use information about psychological and pharmacological treatment of mental disorders in general practitioner care, despite the fact that most patients with mental health conditions in Sweden are treated by a general practitioner [45]. Such information could have been of value. A limitation with the strategy of focusing on hospital-based mental health care is also that the actual number of participants who receive such specialized care is relatively small. Another limitation is that a major proportion of adults suffering from mental disorders does not seek or receive adequate treatment. Help-seeking behavior is lower among men than among women, and untreated mental disorders are not uncommon [46]. Hence, it is likely that there are individuals captured in our data who suffer from severe mental disorders without having received hospital-based treatment. This might result in an underestimation of the actual need of adult hospital-based care. Furthermore, the data on hospital-based outpatient care did not include all registered cases, which implies an underestimation of the total use of hospital-based care and a higher weight of in-patient care compared to out-patient care. Still it seems unlikely that the general findings in relation to our research questions would be affected.

Finally, we lack data on specific somatic diagnoses in adolescence. Hence, we were not able to disentangle whether the association between somatic symptoms in adolescence and hospital-based mental health care in adulthood was due to somatic symptoms with or without a medical explanation. While we did include a measure of hospitalization due to somatic illness or injury in adolescence, this variable might have captured only a portion of the adolescents with somatic illness. Another limitation with this measure is that it was based on adolescents’ self-reports.

## Conclusions

Somatic symptoms in adolescence predicted severe mental illness in adulthood as measured by hospital-based care. The prediction remained significant even when adjusted for sex, adolescent depression and anxiety, and other confounders. The presence of at least one somatic symptom compared to none in adolescence was shown to be the strongest predictor of future inpatient care due to mood disorders, surpassing sex, adolescent depression, and anxiety. The findings indicate that adolescents with somatic symptoms need early treatment and extended follow-up due to the increased risk of subsequent poor mental health outcomes.

## Additional file

**Additional file 1: Appendix S1.** Frequencies of specific somatic symptoms and differences between individuals without and with adolescent depression. **Appendix S2.** Frequencies of any hospital-based mental health care diagnosis by specific somatic symptoms.

## Abbreviations

DICA-R-A: Diagnostic Interview for Children and Adolescents-Revised-Adolescent
DSM-III-R: Diagnostic and Statistical Manual of Mental Disorders-III-Revised
DSM-IV: Diagnostic and Statistical Manual of Mental Disorders-IV
DSM-5: Diagnostic and Statistical Manual of Mental Disorders-5
HPA: hypothalamic–pituitary–adrenal
ICD-10: International Classification of Diseases-10
MDD: major depressive disorder
SCI: Somatic Symptom Checklist Instrument
