# Supplementary material for: Somatic symptoms in adolescence as a predictor of severe mental illness in adulthood: a long-term community-based follow-up study
Source: Child Adolesc Psychiatry Ment Health. 2018 Aug 14;12:42. doi: 10.1186/s13034-018-0245-0 (PMC6090675; doi:10.1186/s13034-018-0245-0)
Supplement: Supplementary file 1 — Additional file 1: Appendix S1. Frequencies of specific somatic symptoms and differences between individuals without and with adolescent depression. Appendix S2. Frequencies of any hospital-based mental health care diagnosis by specific somatic symptoms. [file 13034_2018_245_MOESM1_ESM.docx]

**Appendix S1. Frequencies of specific somatic symptoms and differences between individuals without and with adolescent depression.**

|  | All  (n=375) | Individuals without adolescent depression  (n=182) | Individuals with adolescent depression  (n=193) |  |  |
| --- | --- | --- | --- | --- | --- |
|  |  |  |  |  |  |
|  | % (n) | % (n) | % (n) | OR | 95% CI |
| Tiredness | 44.0 (165) | 27.5 (50) | 59.6 (115) | 3.89*** | 2.52-6.01 |
| Headache | 26.1 (98) | 12.1 (22) | 39.4 (76) | 4.72*** | 2.78-8.03 |
| Feeling chilly | 23.7 (89) | 14.8 (27) | 32.1 (62) | 2.72*** | 1.63-4.52 |
| Insomnia | 18.9 (71) | 8.2 (15) | 29.0 (56) | 4.55*** | 2.47-8.40 |
| Eye tiredness | 14.7 (55) | 8.2 (15) | 20.7 (40) | 2.91** | 1.55-5.48 |
| Abdominal pain | 11.7 (44) | 5.5 (10) | 17.6 (34) | 3.68** | 1.76-7.69 |
| Dizziness | 8.8 (33) | 3.9 (7) | 13.5 (26) | 3.89** | 1.65-9.21 |
| Nausea | 8.8 (33) | 3.3 (6) | 14.0 (27) | 4.77** | 1.92-11.85 |
| Perspiration | 8.8 (33) | 6.6 (12) | 10.9 (21) | 1.73 | 0.83-3.63 |
| Appetite problem | 8.5 (32) | 2.8 (5) | 14.0 (27) | 5.76*** | 2.17-15.30 |
| Breathing problem | 5.3 (20) | 2.8 (5) | 7.8 (15) | 2.98* | 1.06-8.38 |
| Polyuria | 4.3 (16) | 2.8 (5) | 5.7 (11) | 2.14 | 0.73-6.28 |
| Limb pain | 4.3 (16) | 3.9 (7) | 4.7 (9) | 1.22 | 0.45-3.35 |
| Itching | 4.0 (15) | 1.7 (3) | 6.2 (12) | 3.96* | 1.10-14.25 |
| Dry mouth | 4.0 (15) | 3.9 (7) | 4.2 (8) | 1.08 | 0.38-3.04 |
| Palpitation | 2.9 (11) | 1.1 (2) | 4.7 (9) | 4.40 | 0.94-20.66 |
| Constipation | 1.9 (7) | 1.1 (2) | 2.6 (5) | 2.39 | 0.46-12.49 |
| Fainting | 1.6 (6) | 0.0 (0) | 3.1 (6) | - | - |
| Chewing pain | 1.3 (5) | 1.7 (3) | 1.0 (2) | 0.62 | 0.10-3.78 |
| Regurgitation | 1.3 (5) | 0.0 (0) | 2.6 (5) | - | - |
| Swallowing problems | 1.1 (4) | 1.7 (3) | 0.5 (1) | 0.31 | 0.03-3.01 |

***p<0.001 **p<0.01 *p<0.05

**Appendix S2. Frequencies of any hospital-based mental health care diagnosis by specific somatic symptoms.**

|  | Any  hospital-based mental health care diagnosis (n=375) | |  |  |
| --- | --- | --- | --- | --- |
|  | Somatic symptom not recorded | Somatic symptom recorded |  |  |
|  | % (n) | % (n) | OR | 95% CI |
| Tiredness | 11.0 (23) | 20.6 (34) | 2.11* | 1.19-3.75 |
| Headache | 13.0 (36) | 21.4 (21) | 1.83* | 1.01-3.31 |
| Feeling chilly | 13.3 (38) | 21.4 (19) | 1.77 | 0.96-3.26 |
| Insomnia | 12.8 (39) | 25.4 (18) | 2.31** | 1.23-4.34 |
| Eye tiredness | 14.7 (47) | 18.2 (10) | 1.29 | 0.61-2.74 |
| Abdominal pain | 13.6 (45) | 27.3 (12) | 2.38* | 1.14-4.97 |
| Dizziness | 14.3 (49) | 24.2 (8) | 1.91 | 0.82-4.48 |
| Nausea | 13.2 (45) | 36.4 (12) | 3.77** | 1.74-8.19 |
| Perspiration | 14.0 (48) | 27.3 (9) | 2.30* | 1.01-5.24 |
| Appetite problem | 14.6 (50) | 21.9 (7) | 1.64 | 0.67-4.00 |
| Breathing problem | 14.4 (51) | 30.0 (6) | 2.55 | 0.94-6.95 |
| Polyuria | 14.8 (53) | 25.0 (4) | 1.92 | 0.60-6.19 |
| Limb pain | 14.2 (51) | 37.5 (6) | 3.62* | 1.26-10.40 |
| Itching | 14.7 (53) | 26.7 (4) | 2.11 | 0.65-6.86 |
| Dry mouth | 15.0 (54) | 20.0 (3) | 1.42 | 0.39-5.19 |
| Palpitation | 14.6 (53) | 36.4 (4) | 3.35 | 0.95-11.85 |
| Constipation | 15.2 (56) | 14.3 (1) | 0.93 | 0.11-7.86 |
| Fainting | 15.2 (56) | 16.7 (1) | 1.12 | 0.13-9.75 |
| Chewing pain | 15.1 (56) | 20.0 (1) | 1.40 | 0.15-12.77 |
| Regurgitation | 14.9 (55) | 40.0 (2) | 3.82 | 0.62-23.38 |
| Swallowing problems | 15.4 (57) | 0.0 (0) | - | - |

***p<0.001 **p<0.01 *p<0.05
